# Transcriptomic data from two primary cell models stimulating human monocytes suggest inhibition of oxidative phosphorylation and mitochondrial function by *N. meningitidis* which is partially up-regulated by IL-10

**DOI:** 10.1186/s12865-017-0229-5

**Published:** 2017-10-27

**Authors:** Unni Gopinathan, Reidun Øvstebø, Berit Sletbakk Brusletto, Ole Kristoffer Olstad, Peter Kierulf, Petter Brandtzaeg, Jens Petter Berg

**Affiliations:** 1 Blood Cell Research Group, Section for Research, Department of Medical Biochemistry, Oslo University Hospital, Oslo, Norway; 2 Department of Pediatrics, Oslo University Hospital, Oslo, Norway; 3 Institute of Clinical Medicine, Faculty of Medicine, University of Oslo, Oslo, Norway

**Keywords:** Gene expression, mRNA, Bioinformatics, Meningococcal sepsis, *N. Meningitidis*, Interleukin-10, Ingenuity pathway analysis, Gene set enrichment analysis

## Abstract

**Background:**

Biological interpretation of DNA microarray data may differ depending on underlying assumptions and statistical tests of bioinformatics tools used. We used Gene Set Enrichment Analysis (GSEA) and Ingenuity Pathway Analysis (IPA) to analyze previously generated DNA microarray data from human monocytes stimulated with *N. meningitidis* and IL-10 (“the model system”), and with meningococcal sepsis plasma before and after immunodepletion of IL-10 (“the patient plasma system”). The objectives were to compare if the two bioinformatics methods resulted in similar biological interpretation of the datasets, and to identify whether GSEA provided additional insight compared with IPA about the monocyte host response to meningococcal activation.

**Results:**

In both experimental models, GSEA and IPA identified genes associated with pro-inflammatory innate immune activation, including TNF-signaling, Toll-like receptor signaling, JAK-STAT-signaling, and type I and type II interferon signaling. GSEA identified genes regulated by the presence of IL-10 with similar gene sets in both the model system and the patient plasma system. In the model system, GSEA and IPA in sum identified 170 genes associated with oxidative phosphorylation/mitochondrial function to be down-regulated in monocytes stimulated with meningococci. In the patient plasma system, GSEA and IPA in sum identified 122 genes associated with oxidative phosphorylation/mitochondrial dysfunction to be down-regulated by meningococcal sepsis plasma depleted for IL-10. Using IPA, we identified IL-10 to up-regulate 18 genes associated with oxidative phosphorylation/mitochondrial function that were down-regulated by *N. meningitidis*.

**Conclusions:**

Biological processes associated with the gene expression changes in the model system of meningococcal sepsis were comparable with the results found in the patient plasma system. By combining GSEA with IPA, we discovered an inhibitory effect of *N. meningitidis* on genes associated with mitochondrial function and oxidative phosphorylation, and that IL-10 partially reverses this strong inhibitory effect, thereby identifying, to our knowledge, yet another group of genes where IL-10 regulates the effect of LPS. We suggest that relying on a single bioinformatics tool together with an arbitrarily chosen filtering criteria for data analysis may result in overlooking relevant biological processes and signaling pathways associated with genes differentially expressed between compared experimental conditions.

**Electronic supplementary material:**

The online version of this article (10.1186/s12865-017-0229-5) contains supplementary material, which is available to authorized users.

## Background

Sepsis, defined as life-threatening organ dysfunction caused by a dysregulated host response to infection [1], remains a serious cause of mortality in intensive care units worldwide [2, 3]. The past decade has seen a large number of studies aiming to investigate the pathophysiology of sepsis through systems biology approaches, with genome-wide expression profiling being a widely used method [4–6]. This approach has in part been motivated by the failure of a large number of clinical trials directed towards the specific inhibition of endotoxin and cytokines [7, 8], and the need to increase our understanding of the complexity of the sepsis syndrome [9, 10].

Meningococcal sepsis is an overwhelming form of the sepsis syndrome, with previous studies identifying mortality within 12–24 h in previously healthy children and adults [11]. The intense and rapidly evolving pro-inflammatory response resulting from high levels of meningococci and lipopolysaccharides (LPS, endotoxin) in blood [12–16] is suggested to be the main determinant of septic shock with multi-organ failure, coagulopathy and mortality. Our research group has over the past two decades used a primary cell model consisting of elutriated and cryopreserved human monocytes to elucidate cellular mechanisms activated by meningococcal sepsis plasma [14, 17–20]. We have viewed human monocytes as a relevant cellular model for several reasons, including their pro-coagulant activity in meningococcal sepsis [21, 22], and their role as phagocytic cells in various tissues after differentiation to macrophages [23].

Our group has recently investigated the gene expression profile induced by *N. meningitidis* in human monocytes. The first study demonstrated that *N. meningitidis* and purified LPS can differentially regulate the expression of over 4600 genes [18]. Follow-up studies have investigated the differential expression of genes induced by plasma from patients with meningococcal sepsis, with a special focus on the biological significance of the anti-inflammatory cytokine IL-10. The highest concentrations of IL-10 are found in non-survivors of meningococcal septic shock [16, 17, 24]. We have studied the effect of IL-10 in two previous studies, one stimulating monocytes with wild-type *N. meningitidis* [25] and recombinant IL-10, and the other using plasma samples from patients with severe meningococcal sepsis or septic shock [26]. In both studies we showed that IL-10 regulates a group of genes that are induced by *N. meningitidis*, and that these genes are associated with a broad range of functional categories.

Over a decade ago, the main approach to gene expression analysis was to focus on genes representing the largest difference between two experimental conditions, namely those at the top and bottom of a list. However, as described by Subramanian et al. [27], this approach has had a number of limitations. First, an extremely large list may make selection of relevant genes daunting. Secondly, individual genes may not meet the threshold for statistical significance once correction for multiple testing is performed. Finally, focusing on individual genes may overlook important effects suggested by a set of genes that are coordinately expressed, but where the fold change (FC) level does not exceed the cut-off point. It is in response to these challenges that gene set enrichment analysis (GSEA), as initially described by Mootha et al. [28] and later developed by Subramanian et al. [27], was established.

Our previous gene expression studies have used the commercially available tool, Ingenuity Pathway Analysis (IPA) for data analysis [29]. The IPA software analyzes the gene expression profile against molecular relationships in the Ingenuity Knowledge Base, which is a repository of functional annotations, biological interactions and modelled relationships between proteins, genes, cells, tissues, drugs and diseases reported from primary literature sources, including peer-reviewed journal articles and review articles, and other databases (e.g., KEGG, EntrezGene, the Gene Ontology Project). IPA has become among the most widely used tools for gene expression analysis. Similar to GSEA, IPA is a form of enrichment analysis, which is an analytic strategy aiming to identify whether a group of genes is significantly associated with a particular biological process or signaling pathway [30]. GSEA and IPA differ with respect to the statistical approach taken to identifying the significantly enriched groups of genes [27, 31].

Over time, the increasing number of bioinformatics tools have made it easier for investigators to make biological interpretation of genes differentially expressed between different experimental conditions. However, the increasing number of tools have also made comparisons between gene expression studies, such as those for sepsis [5], more difficult, since biological interpretation may differ depending on the methods’ underlying assumptions and statistical tests. One approach to overcome these challenges is to expose the dataset to two different data analytical methods, and consider whether the methods yield similar biological interpretation. In this study we used IPA and GSEA to analyze previously generated DNA microarray data from studies of human monocyte host response to *N. meningitidis*, IL-10 and meningococcal sepsis plasma [25, 26]. The objectives were to compare whether these two methods resulted in similar biological interpretation of the datasets; and identify whether GSEA provided additional insight about the human monocyte host response to meningococcal activation.

## Methods

### Definitions of the compared experimental conditions and models

Gene expression data from two different, previously published models of transcriptomic changes induced in human monocytes were used for this study (Table 1). The data and protocols of both studies are compliant with the minimum information about a microarray experiment (MIAME) guidelines [32]. In the first model [25], gene expression changes were induced in human monocytes stimulated with the *N. meningitidis* reference strain 44/76 and recombinant IL-10. The control group was unstimulated human monocytes. This model is hereafter denoted the ***“the model system”***. In the second model [26] gene expression changes were induced in human monocytes stimulated with plasma from patients with severe meningococcal sepsis or septic shock containing high (≥130 endotoxin units (EU)/mL) levels of LPS, before and after immunodepletion of native IL-10. ***Plasma samples with IL-10*** are denoted ***“patient plasma with IL-10”.*** Plasma samples after IL-10 immunodepletion are denoted ***“IL-10 immunodepleted patient plasma”***. The control group was human monocytes stimulated with plasma from non-shock patients with meningococcal meningitis or mild meningococcemia, containing low (≤1.4 EU/mL) levels of LPS and IL-10. These plasma samples are as a group denoted ***“low LPS plasma”***. The clinical course, microbiological data and cytokine measurements of each patient have previously been reported [26]. This model is hereafter denoted the **“** ***The patient plasma system*** **”.** In both models the monocytes were stimulated for three hours. This study compared results between three experimental conditions in each model system. In ***the model system*** the following conditions were compared: monocytes stimulated with *N. meningitidis* versus unstimulated monocytes (***Nm*** **vs** ***ctr*** **)**, monocytes stimulated with *N. meningitidis* in combination with IL-10 versus unstimulated monocytes (***Nm + IL-10*** **vs** ***ctr***), and monocytes stimulated with *N. meningitidis* and IL-10 versus monocytes stimulated with *N. meningitidis* only (***Nm + IL-10*** **vs** ***Nm*** **)**. In ***the patient plasma system*** the following experimental conditions were compared: **IL-10 immunodepleted plasma vs low LPS plasma, patient plasma with IL-10 vs low LPS plasma,** and **IL-10 immunodepleted plasma vs patient plasma with IL-10**.

**Table 1:** Experimental conditions from which DNA microarray data was generated

In vitro experimental model	Experimental conditions			
Model system	Unstimulated (“Ctr”)	10^6^/mL *N. meningitidis* (“Nm”)	10^6^/mL *N. meningitidis* + 25 ng/mL IL-10 (“Nm + IL-10”)	25 ng/mL IL-10 (“IL-10”)^a^
Patient plasma system	Plasma from patients with meningococcal meningitis or mild meningococcemia (“low LPS plasma”)	Plasma from patients with severe meningococcal sepsis or septic shock, depleted for IL-10 (“IL-10 immunodepleted plasma”)	Plasma from patients with severe meningococcal sepsis or septic shock (“patient plasma with IL-10”)	Plasma from patients with meningococcal meningitis or mild meningococcemia, depleted for IL-10 (“low LPS plasma immunodepleted for IL-10”)^a^

^a^Data from these experimental conditions were not used in the present study

### Gene set enrichment analysis

Gene set enrichment analysis (GSEA) [27, 28] was performed using GSEA version 2.0.14 [33]. Data from previously generated gene expression profiles induced in *the model system* [25] and in *the patient plasma system* [26] were imported into the GSEA software. Normalized and background corrected log_2_-transformed signal intensities after using the Robust Multichip Analysis (RMA) algorithm implemented in Partek Genomic Suit software were used. For this study, we analyzed the expression profiles against the hallmark gene sets available from the Molecular Signatures Database [34]. The hallmark gene sets are *a priori* defined genes that have been identified by computational methods to be coordinately expressed in various biological states and processes. GSEA was run according to default parameters: probes for the same gene were collapsed into a single gene symbol (identified by its HUGO gene symbol), permutation number was set to 1000, and permutation type was set to “gene sets.” The conventional cut-off value for statistical significance used in GSEA is a false discovery rate (FDR) of 25%. In order to reduce the likelihood of false positive results, this study used FDR 5% as cut-off value for enriched gene sets.

### Ingenuity pathway analysis

Gene lists (Excel files) containing gene identifiers (probe set IDs), and corresponding *P*-values were uploaded to Ingenuity Pathway Analysis (IPA, Ingenuity Systems, www.ingenuity.com). A cut-off at FDR <5% was set to identify significantly differentially expressed genes. Several tools in IPA were used to analyze the gene expression data. The canonical pathway tool was used to identify the top canonical pathways associated with the genes differentially expressed between compared conditions. Biological functions associated with the differentially expressed genes were identified by mapping each gene to its corresponding function in the Ingenuity Knowledge Base. The right-tailed Fisher’s exact test was performed in IPA to calculate a *P-*value determining the probability that each biological function assigned to the data set was due to chance alone. The *P*-value was corrected for multiple comparisons using the Benjamini-Hochberg method for correcting the FDR [35]. Permission was granted by QIAGEN Silicon Valley to use copyrighted figures generated from Ingenuity Pathways Analysis in this publication. Figures produced from IPA are available under an open-access CC-BY license for purposes of publication.

### Canonical pathway analysis in IPA

There are two main groups of canonical pathways in IPA – metabolic and signaling – and these are hierarchically grouped according to a number of sub-categories (Panel 1). In order to identify those pathways most relevant to the experimental condition and disease of study, disease- and cell-specific pathways not deemed relevant to the experimental conditions under study were excluded from the analysis. Canonical pathways significantly enriched by the differentially expressed genes in the datasets were identified with the right-tailed Fisher’s Exact Test, which calculates a *P-*value determining the probability that the canonical pathway is associated with the data set due to random chance alone. The *P-*values were corrected for multiple testing using the Benjamini-Hochberg method for correcting the false discovery rate [31].

### Statistical analysis

The GSEA methodology calculates three statistically important values. The first is the *enrichment score*, which reflects the degree to which an a priori defined list of genes *S* is overrepresented in a ranked list of genes *L*. In this study, the *a priori* defined list of genes *S* are the hallmark gene sets described above, and the list *L* are genes ranked according to log_2_-transformed signal intensities from each experimental condition studied. The enrichment score is calculated by walking down the list *L*, increasing a running-sum statistic when a gene in S is encountered, and decreasing it when encountering a gene not in *S.* The magnitude of the increment depends on the correlation of the genes with the phenotype. The enrichment score is the maximum deviation from zero encountered in the random walk, and corresponds to a weighted Kolmogorov–Smirnov-like statistic, as described more extensively in the supplementary material by Subramanian et al. [27]. The second is *the nominal P value* which is calculated to estimate the significance of the enrichment score. The nominal *P* value is then normalized to account for the size of the gene set, and then adjusted for multiple testing by calculating the *false discovery rate (FDR)*, which is the third important statistical value. The FDR estimates the probability that a given enrichment score represents a false positive finding. The threshold for statistical significance was set to FDR 5%. The differentially expressed genes for IPA were identified using two-way ANOVA in Partek Genomic Suite, as previously described [25, 26]. In the present study, *P*-values were corrected using the Benjamini-Hochberg method [35] for controlling the FDR, and a FDR of <5% was set as requirement for inclusion in the analysis. No FC-value was set as cut-off.

## Results

### GSEA and IPA associated gene expression induced in *the model system* with similar sets of signaling pathways

We first examined the gene expression changes induced in *the model system*, beginning with GSEA. The enriched gene sets used in GSEA represent genes that previous genome-wide expression studies have demonstrated to be coordinately expressed in different experimental conditions and biological states [34]. In ***Nm*** **vs** ***ctr***, we identified 15 hallmark gene sets to be significantly enriched (FDR <5%) (Table 2). Twelve of these 15 gene sets were significantly enriched in ***Nm + IL-10*** **vs** ***ctr*** (Table 3), only differing with respect to the gene sets epithelial mesenchymal transition, coagulation, and estrogen response late which no longer were significantly expressed.

**Table 2:** Gene sets enriched in human monocytes stimulated with *N. meningitidis* vs control

Name of hallmark gene set	Description^a^	FDR	Number of genes in the gene set	Founder gene sets^b^
Interferon alpha response	Genes up-regulated in response to alpha interferon proteins	<0.001	97	82
Interferon gamma response	Genes up-regulated in response to IFNG	<0.001	200	82
TNF signaling via NfkB	Genes regulated by NF-kB in response to TNF	<0.001	200	120
Inflammatory response	Genes defining inflammatory response	<0.001	200	120
Allograft rejection	Genes up-regulated during transplant rejection	<0.001	200	190
IL6 JAK-STAT3 signalling	Genes up-regulated by IL6 [via STAT3, e.g., during acute phase response]	<0.001	87	24
IL2 STAT5 signaling	Genes up-regulated by STAT5 in response to IL2 stimulation	<0.001	200	13
KRAS signaling down	Genes down-regulated by KRAS activation	<0.001	200	16
KRAS signaling up	Genes up-regulated by KRAS activation	<0.001	200	14
Apoptosis	Genes mediating programmed cell death (apoptosis) by activation of caspases	<0.001	161	80
Complement	Genes encoding components of the complement system, which is part of the innate immune system	0.001	200	71
Epithelial mesenchymal transition	Genes defining epithelial-mesenchymal transition, as in wound healing, fibrosis and metastasis	0.001	200	106
Coagulation	Genes encoding components of blood coagulation system; also up-regulated in platelets	0.015	138	71
Estrogen response late	Genes defining late response to estrogen	0.007	200	59
Hypoxia	Genes up-regulated in response to low oxygen levels (hypoxia)	0.015	200	87

^a^Description of the gene sets were obtained by the Molecular Signatures Database

^b^Founder gene sets constitute the original experiments associating the genes with specified functions or biological processes

**Table 3:** Gene sets enriched in human monocytes stimulated with *N. meningitidis* and IL-10 vs control

Name of hallmark gene set	Description^a^	FDR	Number of genes in the gene set	Founder gene sets^b^
Interferon gamma response	Genes up-regulated in response to IFNG	<0.001	200	82
Interferon alpha response	Genes up-regulated in response to alpha interferon proteins.	<0.001	97	82
TNF signaling via NfkB	Genes regulated by NF-kB in response to TNF	<0.001	200	120
Inflammatory response	Genes defining inflammatory response	<0.001	200	120
IL2 STAT5 signaling	Genes up-regulated by STAT5 in response to IL2 stimulation.	<0.001	200	13
IL6 JAK-STAT3 signaling	Genes up-regulated by IL6 [via STAT3, e.g., during acute phase response]	<0.001	87	24
Allograft rejection	Genes up-regulated during transplant rejection	<0.001	200	190
Complement	Genes encoding components of the complement system, which is part of the innate immune system	<0.001	200	71
KRAS signaling up	Genes up-regulated by KRAS activation	0.003	200	14
KRAS signaling down	Genes down-regulated by KRAS activation	0.007	200	16
Apoptosis	Genes mediating programmed cell death (apoptosis) by activation of caspases	0.007	161	80
Hypoxia	Genes up-regulated in response to low oxygen levels (hypoxia)	0.039	200	87

^a^Description of the gene sets were obtained from the Molecular Signatures Database

^b^Founder gene sets constitute the original experiments associating the genes with specified functions or biological processes

**Fig. 1:**
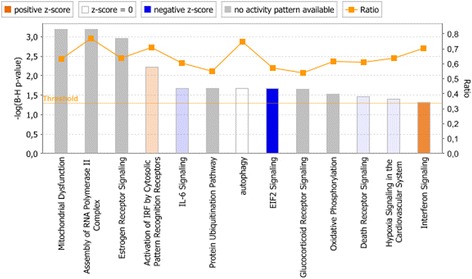
Canonical pathways significantly enriched in human monocytes when comparing Nm vs ctr. Significantly enriched canonical pathways were identified with a right-tailed Fisher’s Exact Test that calculates a *P-*value determining the probability that each canonical pathway associated with the dataset was due to chance alone. The *P-*values were corrected for multiple testing using the Benjamini-Hochberg method for correcting the FDR. The z-score indicates predicted activation state of the canonical pathway. Blue color or lighter shades of blue indicate a negative z-score and down-regulation of the pathway, and orange color or lighter shades of orange indicate a positive z-score and up-regulation of the pathway. Ratio denotes the number of significantly expressed genes compared with the total number of genes associated with the canonical pathway

**Fig. 2:**
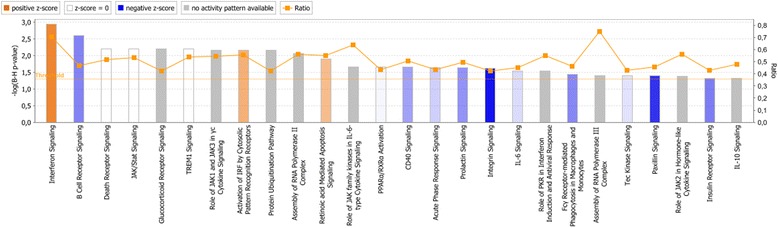
Canonical pathways significantly enriched in human monocytes when comparing Nm + IL-10 vs ctr. Significantly enriched canonical pathways were identified with a right-tailed Fisher’s Exact Test that calculates a *P-*value determining the probability that each canonical pathway associated with the dataset was due to chance alone. The *P-*values were corrected for multiple testing using the Benjamini-Hochberg method for correcting the FDR. The z-score indicates predicted activation state of the canonical pathway. Blue color or lighter shades of blue indicate a negative z-score and down-regulation of the pathway, and orange color or lighter shades of orange indicate a positive z-score and up-regulation of the pathway. Ratio denotes the number of significantly expressed genes compared with the total number of genes associated with the canonical pathway

The significantly enriched gene sets in each experimental condition were then compared against significantly enriched canonical pathways in IPA. In ***Nm*** **vs** ***ctr*** (Fig. 1), both GSEA and IPA identified differentially expressed genes to be associated with apoptosis (denoted “Death Receptor Signaling” by IPA), hypoxia (in IPA denoted “Hypoxia in the Cardiovascular System”), interferon signaling and IL-6 signaling. In ***Nm + IL-10*** **vs** ***ctr*** (Fig. 2), both GSEA and IPA identified apoptosis, interferon signaling, and JAK-STAT signaling to be affected. A greater number of canonical pathways were significantly enriched in monocytes stimulated with *N. meningitidis* together with IL-10 than with *N. meningitidis* alone, indicating the biological effect induced by the presence of IL-10.

### GSEA and IPA associated gene expression induced in *the patient plasma system* with similar sets of signaling pathways

We next used GSEA and IPA to examine the gene expression profiles induced in ***the patient plasma system***. The GSEA findings (Tables 4 and 5) identified enrichment of biologically relevant gene sets similar to those in ***the model system***, including those related to inflammatory response, interferon response, JAK-STAT signaling, TNF signaling, IL-6 signaling and IL-2 signaling, complement, apoptosis and hypoxia. These signaling pathway and biological processes were identified to be regulated in both experimental conditions (***patient plasma with IL-10*** **vs** ***low LPS plasma***, and ***IL-10 immunodepleted plasma*** **vs** ***low LPS plasma*** **)**.

**Table 4:** Gene sets enriched in human monocytes stimulated with IL-10 immunodepleted plasma vs low LPS plasma

Name of hallmark gene set	Description^a^	FDR	Number of genes in the gene set	Founder gene sets^b^
TNF signaling via NfkB	Genes regulated by NF-kB in response to TNF	<0.001	200	120
Inflammatory response	Genes defining inflammatory response.	<0.001	200	120
Interferon gamma response	Genes up-regulated in response to IFNG	<0.001	200	82
Interferon alpha response	Genes up-regulated in response to alpha interferon proteins.	<0.001	97	82
IL2 STAT5 signaling	Genes up-regulated by STAT5 in response to IL2 stimulation.	<0.001	200	13
IL6 JAK-STAT3 signaling	Genes up-regulated by IL6 [via STAT3, e.g., during acute phase response.	<0.001	87	24
KRAS signaling up	Genes up-regulated by KRAS activation.	0.001	200	14
Epithelial mesenchymal transition	Genes defining epithelial-mesenchymal transition, as in wound healing, fibrosis and metastasis	<0.001	200	
Allograft rejection	Genes up-regulated during transplant rejection.	<0.001	200	190
MYC targets_v2	A subgroup of genes regulated by MYC	0.004	58	6
Complement	Genes encoding components of the complement system, which is part of the innate immune system.	0.004	200	71
Coagulation	Genes encoding components of blood coagulation system	0.005	138	71
Apoptosis	Genes mediating programmed cell death (apoptosis) by activation of caspases.	0.016	161	80
Estrogen response early	Genes defining early response to estrogen	0.018	200	
KRAS signaling down	Genes down-regulated by KRAS activation.	0.029	200	16
Hypoxia	Genes up-regulated in response to low oxygen levels (hypoxia).	0.031	200	87
UV response up	Genes up-regulated in response to ultraviolet (UV) radiation	0.044	58	16

^a^Description of the gene sets were obtained from the Molecular Signatures Database

^b^Founder gene sets constitute the original experiments associating the genes with specified functions or biological processes

**Table 5:** Gene sets enriched in human monocytes stimulated with patient plasma with IL-10 vs low LPS plasma

Name of hallmark gene set	Description^a^	FDR	Number of genes in the gene set	Founder gene sets^b^
TNF signaling via NfkB	Genes regulated by NF-kB in response to TNF	<0.001	200	120
Interferon gamma response	Genes up-regulated in response to IFNG	<0.001	200	82
Inflammatory response	Genes defining inflammatory response	<0.001	200	120
Interferon alpha response	Genes up-regulated in response to alpha interferon proteins	<0.001	97	82
IL6 JAK-STAT3 signaling	Genes up-regulated by IL6 [via STAT3, e.g., during acute phase response]	<0.001	87	24
IL2 STAT5 signaling	Genes up-regulated by STAT5 in response to IL2 stimulation	<0.001	200	13
MYC targets_v2	A subgroup of genes regulated by MYC	<0.001	58	6
Epithelial mesenchymal transition	Genes defining epithelial-mesenchymal transition, as in wound healing, fibrosis and metastasis	<0.001		
Complement	Genes encoding components of the complement system	<0.001	200	71
Allograft rejection	Genes up-regulated during transplant rejection	<0.001	200	190
KRAS signaling up	Genes up-regulated by KRAS activation	0.001	200	14
Estrogen response early	Genes defining early response to estrogen	0.001		
Coagulation	Genes encoding components of blood coagulation system	0.001	138	71
UV response up	Genes up-regulated in response to ultraviolet (UV) radiation	0.005	158	16
Hypoxia	Genes up-regulated in response to low oxygen levels (hypoxia)	0.008	200	87
Apoptosis	Genes mediating programmed cell death (apoptosis) by activation of caspases	0.013	161	80
Unfolded protein response	Genes up-regulated during unfolded protein response, a cellular stress response related to the endoplasmic reticulum	0.036	113	22

^a^Description of the gene sets were obtained from the Molecular Signatures Database

^b^Founder gene sets constitute the original experiments associating the genes with specified functions or biological processes

**Fig. 3:**
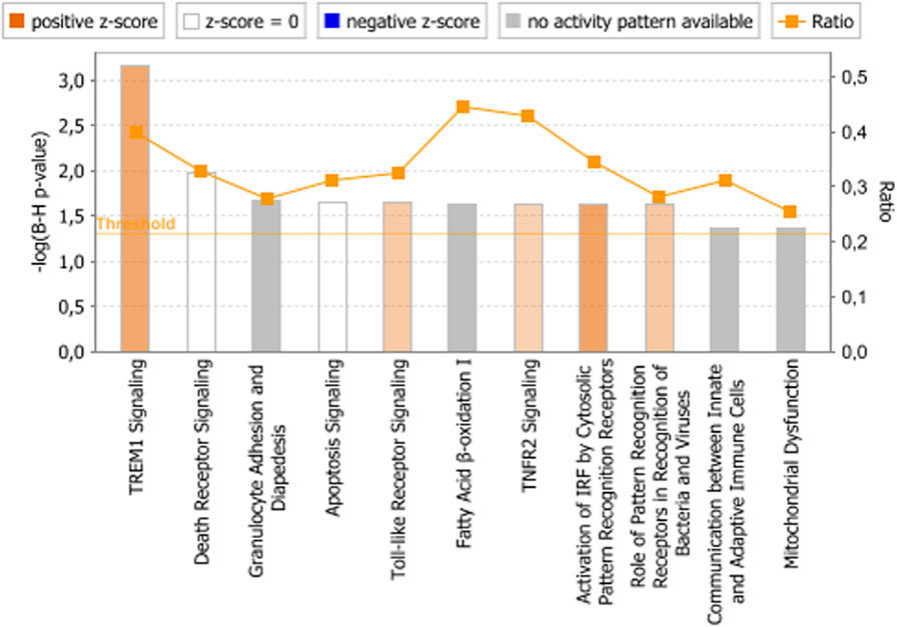
Canonical pathways significantly enriched in human monocytes stimulated with IL-10 immunodepleted plasma vs low LPS plasma. Significantly enriched canonical pathways were identified with a right-tailed Fisher’s Exact Test that calculates a *P-*value determining the probability that each canonical pathway associated with the dataset was due to chance alone. The *P-*values were corrected for multiple testing using the Benjamini-Hochberg method for correcting the FDR. The z-score indicates predicted activation state of the canonical pathway. Blue color or lighter shades of blue indicate a negative z-score and down-regulation of the pathway, and orange color or lighter shades of orange indicate a positive z-score and up-regulation of the pathway. Ratio denotes the number of significantly expressed genes compared with the total number of genes associated with the canonical pathway

**Fig. 4:**
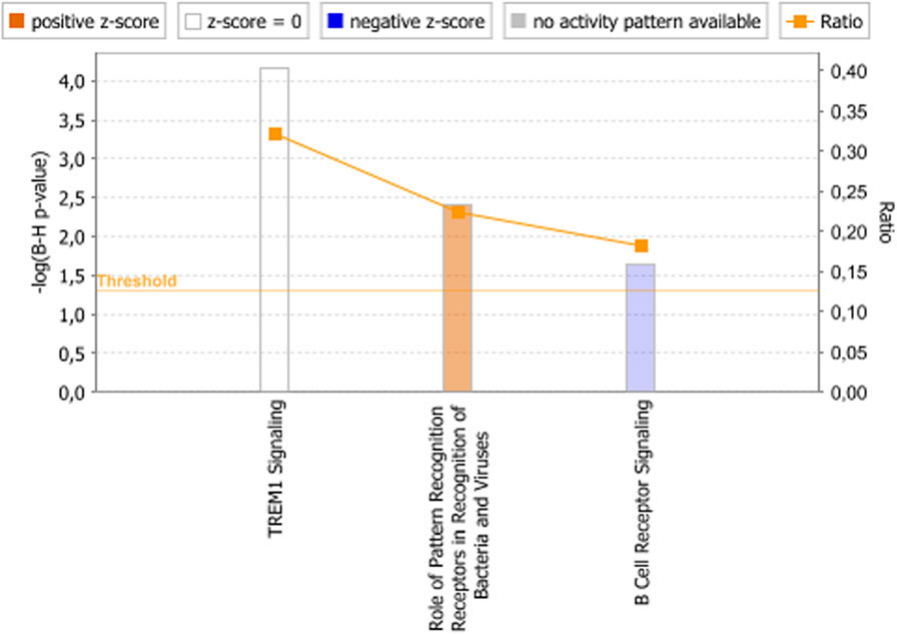
Canonical pathways significantly enriched in human monocytes stimulated with patient plasma with IL-10 vs low LPS plasma. Significantly enriched canonical pathways were identified with a right-tailed Fisher’s Exact Test, that calculates a *P-*value determining the probability that each canonical pathway associated to the dataset was due to chance alone. The *P-*values were corrected for multiple testing using the Benjamini-Hochberg method for correcting the FDR, and a *P*-value of <0.05 was set as threshold for statistical significance. The z-score indicates predicted activation state of the canonical pathway. Blue color or lighter shades of blue indicate a negative z-score and down-regulation of the pathway, and orange color or lighter shades of orange indicate a positive z-score and up-regulation of the pathway. Ratio denotes the number of significantly expressed genes compared with the total number of genes associated with the canonical pathway

We then compared GSEA data with the canonical pathways identified to be significantly enriched by IPA. **IL-10 immunodepleted plasma** induced a significant enrichment of genes associated with apoptosis signaling, TNF-signaling, Toll-like receptor signaling, and interferon response (in IPA identified as “Activation of IRF by Cytosolic Pattern Recognition Receptors”), similar to what was identified by GSEA (Fig. 3). In **patient plasma with IL-10**, IPA only identified the significant enrichment of three canonical pathways, suggesting that the presence of IL-10 had a strong regulatory effect on induction of genes associated with canonical pathways (Fig. 4). However, when we loosened the statistical stringency by setting an uncorrected *P* value of 0.05 as the threshold for significant enrichment of canonical pathways, IPA identified **patient plasma with IL-10** to significantly enrich a larger number of canonical pathways, including IL-6 signaling, JAK/STAT signaling, apoptosis and Toll-like receptor signaling (Additional file 1), as also identified by GSEA.

### Gene set enrichment analysis identified genes differentially induced by IL-10 to be associated with similar gene sets in both *the model system* and *the patient plasma system*

We next used GSEA to investigate the effect of IL-10 on meningococci-induced gene expression in *the model system* and in *the patient plasma system*. In *the model system*, when comparing ***Nm + IL-10*** **vs** ***Nm*** *,* the differentially expressed genes were associated with the significant enrichment of 10 gene sets (Table 6, detailed reports in Additional files 2, 3, 4, 5), with oxidative phosphorylation being the gene set with the highest enrichment score. This indicates that genes associated with oxidative phosphorylation have greater expression levels in monocytes stimulated by *N. meningitidis and IL-10*, than by *N. meningitidis alone*. Comparison of the corresponding experimental conditions in *the patient plasma system* - ***patient plasma with IL-10*** **vs** ***IL-10 immunodepleted plasma*** - identified oxidative phosphorylation to be the most significantly enriched gene set, suggesting that genes associated with oxidative phosphorylation have greater expression in monocytes stimulated with meningococcal LPS together with IL-10 compared with meningococcal LPS alone. In addition, seven of the eight significant gene sets in the model system were identified also in *the patient plasma system*.

**Table 6:** Overview and comparison of significantly enriched gene sets in the model system and the patient plasma system^a^

Gene sets enriched in Nm + IL-10 vs Nm	Gene sets enriched in patient plasma with IL-10 vs IL-10 immunodepleted plasma	Gene sets enriched in Nm vs Nm + IL-10	Gene sets enriched in IL-10 immunodepleted plasma vs patient plasma with with IL-10
Oxidative phosphorylation	Oxidative phosphorylation	TNF signaling	Interferon alpha response
MYC Targets_v1	Peroxisome	KRAS signaling DN	TNF signaling
DNA repair	MYC Targets_v1	Epithelial-mesenchymal transition	UV response
Adipogenesis	Adipogenesis	UV response	Interferon gamma response
Peroxisome	MYC targets_v2	Inflammatory response	Allograft rejection
MYC targets_v2	P53 Pathway	Kras signaling up	Kras signaling up
P53 Pathway	Reactive oxygen species	Apical surface	Hedgehog signaling
Protein secretion	DNA repair	Cholesterol homeostasis	Hallmark angiogenesis
	Fatty Acid Metabolism	Allograft rejection	Mitotic spindle
	Glycolysis	Interferon alpha response	
	Bile acid metabolism	Coagulation	

^a^Detailed reports about the enriched gene sets is available in Additional files 4, 5, 6, 7

**Fig. 5:**
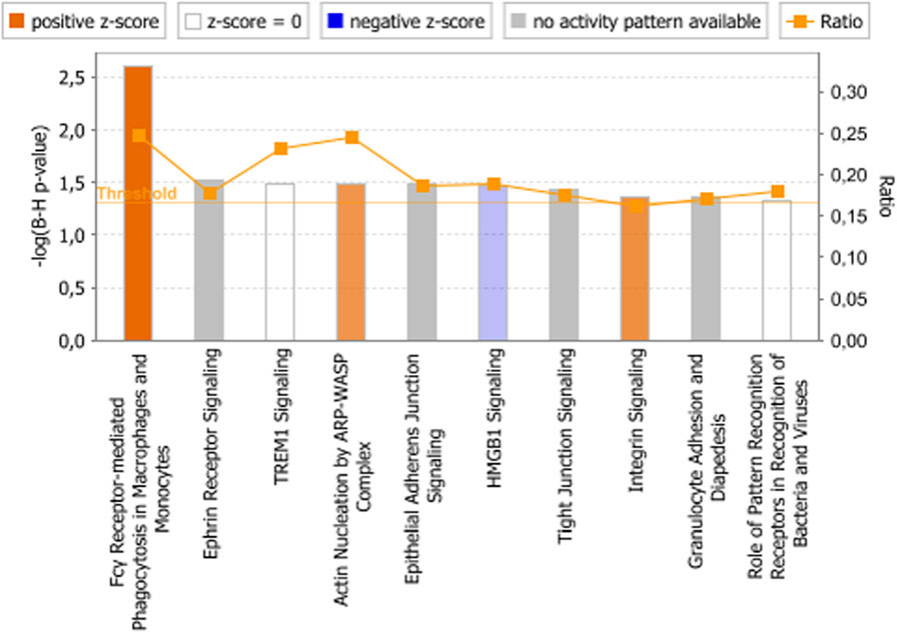
Canonical pathways significantly enriched in human monocytes stimulated with Nm + IL-10 vs Nm^abc^. ^a^Significantly enriched canonical pathways were identified with a right-tailed Fisher’s Exact Test, that calculates a *P-*value determining the probability that each canonical pathway associated to the dataset was due to chance alone. The *P-*values were corrected for multiple testing using the Benjamini-Hochberg method for correcting the FDR. ^b^The z-score indicates predicted activation state of the canonical pathway. Blue color or lighter shades of blue indicate a negative z-score and down-regulation of the pathway, and orange color or lighter shades of orange indicate a positive z-score and up-regulation of the pathway. ^c^Ratio denotes the number of significantly expressed genes compared with the total number of genes associated with the canonical pathway

We next compared gene sets enriched in ***Nm*** **vs** ***Nm + IL-10***. As expected, GSEA identified the enrichment of gene sets associated with the innate immune response, in addition to several other gene sets with probably limited biological relevance to meningococcal infection (such as epithelial-mesenchymal transition, UV response, apical surface, allograft rejection, cholesterol homeostasis). Four of these gene sets (TNF signaling, Interferon alpha response, UV response, Kras signaling up) were significantly enriched when comparing the corresponding experimental conditions in *the patient plasma system* – ***IL-10 immunodepleted*** **vs** ***patient plasma with IL-10***. In comparison, IPA enriched ***Nm + IL-10*** **vs** ***Nm*** (Fig. 5) for biological processes associated with inflammatory signaling (increased activity of TREM1 signaling, and decreased activity of HMGB1 signaling) and cellular integrity and adhesion (including epithelial adherens junction signaling, tight junction signaling, integrin signaling, and granulocyte adhesion and diapedesis). Analysis of ***IL-10 immunodepleted*** **vs** ***patient plasma with IL-10*** did not significantly enrich any biological processes or signaling pathways after Benjamini-Hochberg correction for the false discovery rate (results not shown).

Overall, GSEA analysis suggested that the biological processes associated with the gene expression changes in *the model system* of meningococcal sepsis were comparable with the results found in *the patient plasma system*, but similar biological processes were not enriched in IPA.

### GSEA and IPA identified significant down-regulation of genes associated with mitochondrial function and oxidative phosphorylation in human monocytes stimulated by meningococcal LPS, and partial up-regulation of this response by IL-10

In *the model system*, oxidative phosphorylation was identified by GSEA to be enriched in ***unstimulated monocytes*** **vs** ***monocytes stimulated with N. meningitidis*** (Additional file 6). In the *patient plasma system*, oxidative phosphorylation was the most enriched gene set in ***low LPS plasma*** **vs** ***IL-10 immunodepleted plasma*** (Additional file 7). These two findings suggest that the presence of meningococcal LPS is associated with down-regulation of genes related to oxidative phosphorylation in monocytes.

We therefore examined the association between meningococcal activation of monocytes, and down-regulation of these genes in more detail. In ***IL-10 immunodepleted plasma*** **vs** ***low LPS plasma***, GSEA identified 104 genes to be down-regulated (Additional file 8), while IPA identified 36 genes to be down-regulated (Additional file 9). Eighteen of these genes were identified by both bioinformatics tools. In sum, GSEA and IPA identified 122 genes associated with mitochondrial function/oxidative phosporylation to be down-regulated after depleting meningococcal sepsis plasma for IL-10. When we compared ***Nm*** **vs** ***ctr***, GSEA identified 130 genes to be down-regulated (Additional file 10), and IPA identified 89 genes to be down-regulated (Additional file 11). Here, 49 genes were identified by both tools. Considering the *model system* and the *patient plasma system* together, GSEA and IPA identified 170 genes associated with oxidative phosphorylation and mitochondrial function to be down-regulated in monocytes stimulated by *N. meningitidis* and meningococcal LPS.

**Fig. 6:**
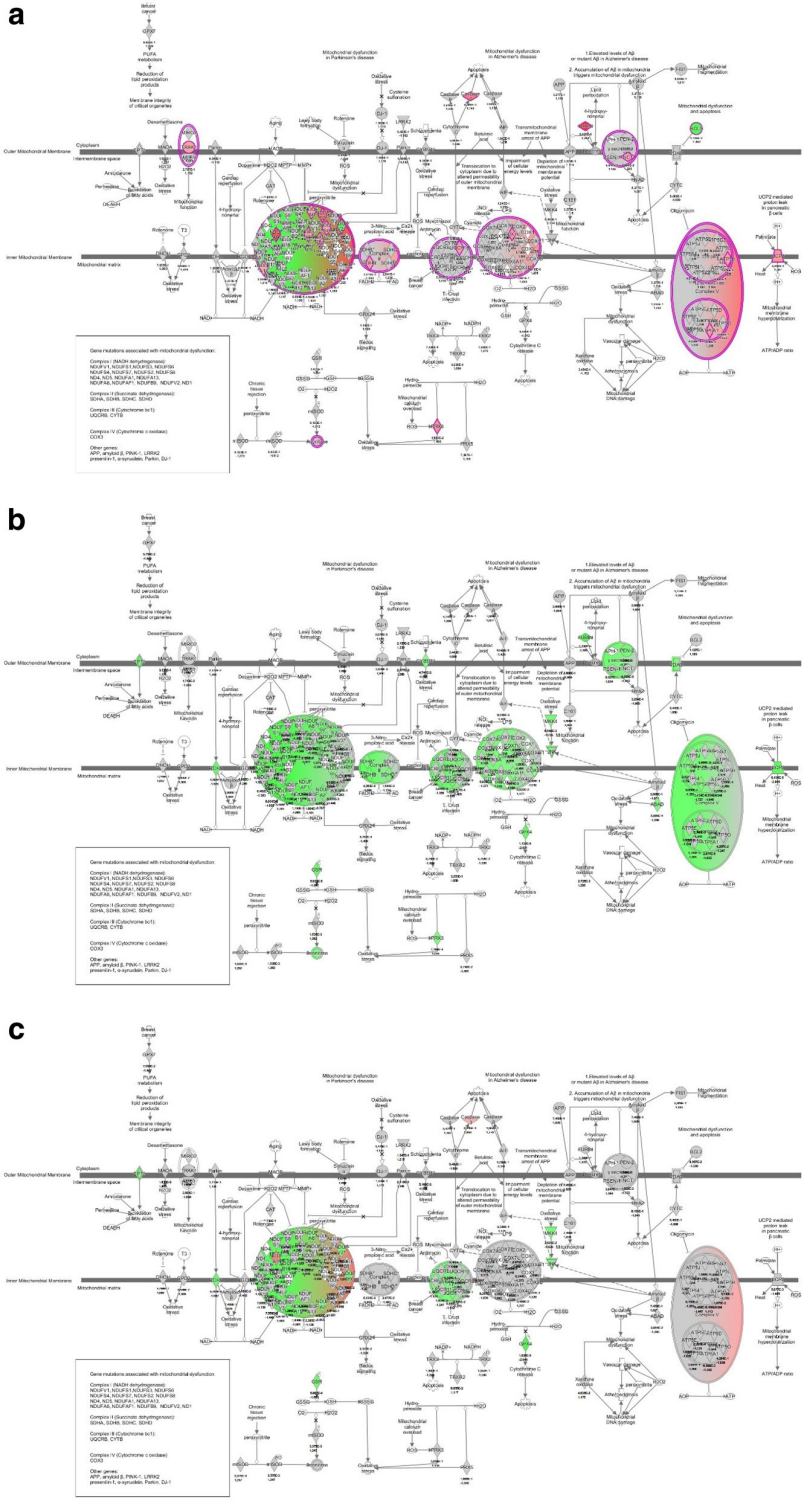
Mitochondrial dysfunction in IPA in human monocytes. **a** Expression levels when comparing *Nm + IL-10 vs Nm*. **b** Expression levels when comparing *Nm* vs *ctr*. **c** Expression levels when comparing *Nm + IL-10 vs ctr*

**Fig. 7:**
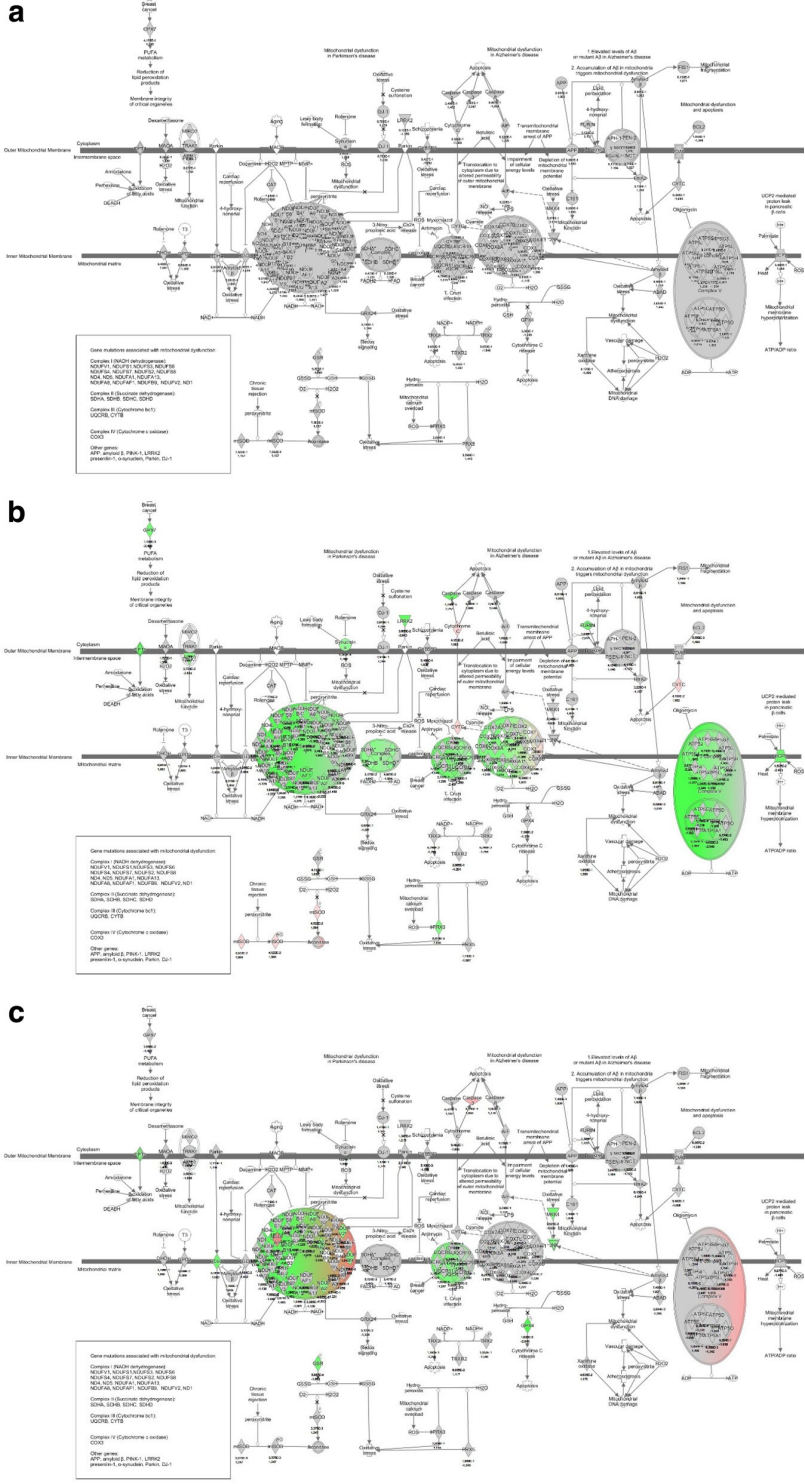
Mitochondrial dysfunction in IPA before and after immunodepletion of IL-10. **a** Expression levels when comparing *patient plasma with IL-10 vs IL-10 immunodepleted plasma.* **b** Expression levels when comparing *patient plasma with IL-10 vs low LPS plasma.* **c** *Expression levels when comparing IL-10 immunodepleted plasma vs low LPS plasma*

Oxidative phosphorylation was also the most enriched gene set when comparing ***Nm + IL-10*** **vs** ***Nm***, and ***patient plasma with IL-10*** **vs** ***IL-10 immunodepleted plasma*** (Table 6), suggesting that the presence of IL-10 up-regulated genes associated with oxidative phosphorylation. To visualize the effect of IL-10 on the Nm-induced down-regulation, we generated the pathway labeled “mitochondrial dysfunction” in IPA, and overlaid *P*-values and FC values calculated from comparing gene expression levels in ***Nm + IL-10*** **vs** ***Nm*** (Fig. 6a). We also overlaid this pathway with *P*-values and FC values generated from comparing ***Nm*** **vs** ***ctr*** (Fig. 6b) and **N** ***m + IL-10*** **vs** ***ctr*** (Fig. 6c). Using IPA, we identified 18 genes to be up-regulated when comparing ***Nm + IL-10*** **vs** ***Nm*** (Table 7), suggesting that the presence of IL-10 partially reversed the down-regulation of genes induced by *N. meningitidis*. We also overlaid *P*-values and FC values generated from comparing experimental conditions in the *patient plasma system* (Fig. 7). Comparing ***patient plasma with IL-10*** **vs** ***IL-10 immunodepleted plasma*** (Fig. 7a) did not identify the presence of IL-10 to significantly up-regulate genes that were down-regulated in IL-10 immunodepleted plasma. However, a greater down-regulation of genes was associated with ***IL-10 immunodepleted plasma*** **vs** ***low LPS plasma*** (Fig. 7b) **than** ***patient plasma with IL-10*** **vs** ***low LPS plasma*** (Fig. 7c). This indicates that the presence of IL-10 in meningococcal sepsis plasma increase the expression of genes associated with mitochondrial function/oxidative phosphorylation**.**

**Table 7:** Genes associated with mitochondrial function identified to be regulated by IL-10 in monocytes stimulated by *N. meningitidis*

Symbol	Entrez gene name	Location	Type(s)	Fold change^1^		
				Nm vs ctr	Nm + IL-10 vs ctr	Nm + IL-10 vs Nm
ACO2	aconitase 2, mitochondrial	Cytoplasm	Enzyme	−1.66	−1.33	1.25
ATP5A1	ATP synthase, H+ transporting, mitochondrial F1 complex, alpha subunit 1, cardiac muscle	Cytoplasm	Transporter	−1.83	−1.34	1.37
ATP5H	ATP synthase, H+ transporting, mitochondrial Fo complex, subunit d	Cytoplasm	Enzyme	−1.50	−1.18	1.27
BCL2	B-cell CLL/lymphoma 2	Cytoplasm	Transporter	NC	NC	−1.46
CASP3	caspase 3, apoptosis-related cysteine peptidase	Cytoplasm	Peptidase	NC	1.86	1.79
COX7C	cytochrome c oxidase subunit VIIc	Cytoplasm	Enzyme	−1.66	NC	1.45
CYB5A	cytochrome b5 type A (microsomal)	Cytoplasm	Enzyme	−1.69	NC	1.48
FURIN	furin (paired basic amino acid cleaving enzyme)	Cytoplasm	Peptidase	−2.47	1.42	3.51
NCSTN	nicastrin	Plasma Membrane	Peptidase	−1.41	1.17	1.21
NDUFA6	NADH dehydrogenase (ubiquinone) 1 alpha subcomplex, 6, 14 kDa	Cytoplasm	Enzyme	−2.00	−1.53	1.31
NDUFA9	NADH dehydrogenase (ubiquinone) 1 alpha subcomplex, 9, 39 kDa	Cytoplasm	Enzyme	1.28	3.10	2.43
NDUFA10	NADH dehydrogenase (ubiquinone) 1 alpha subcomplex, 10, 42 kDa	Cytoplasm	Transporter	−1.38	−1.14	1.22
NDUFAB1	NADH dehydrogenase (ubiquinone) 1, alpha/beta subcomplex, 1, 8 kDa	Cytoplasm	Enzyme	−2.26	−1.56	1.45
NDUFB5	NADH dehydrogenase (ubiquinone) 1 beta subcomplex, 5, 16 kDa	Cytoplasm	Enzyme	−1.48	NC	1.37
NDUFS2	NADH dehydrogenase (ubiquinone) Fe-S protein 2, 49 kDa (NADH-coenzyme Q reductase)	Cytoplasm	Enzyme	−2.04	NC	1.92
NDUFS6	NADH dehydrogenase (ubiquinone) Fe-S protein 6, 13 kDa (NADH-coenzyme Q reductase)	Cytoplasm	Enzyme	−1.74	−1.42	1.22
NDUFV2	NADH dehydrogenase (ubiquinone) flavoprotein 2, 24 kDa	Cytoplasm	Enzyme	NC	−1.61	−1.44
PRDX3	peroxiredoxin 3	Cytoplasm	Enzyme	−1.63	NC	1.47
SDHB	succinate dehydrogenase complex, subunit B, iron sulfur (Ip)	Cytoplasm	Enzyme	−1.91	−1.41	1.35
TRAK1	trafficking protein, kinesin binding 1	Nucleus	Other	−1.29	NC	1.25
UCP2	uncoupling protein 2 (mitochondrial, proton carrier)	Cytoplasm	Transporter	−2.07	−1.49	1.39
UQCR10	ubiquinol-cytochrome c reductase, complex III subunit X	Cytoplasm	Enzyme	−1.75	−1.38	1.26
VPS9D1	VPS9 domain containing 1	Other	Transporter	NC	NC	1.86

^1^Significantly expressed genes identified with two-way ANOVA, *P* < 0.05 after correction for multiple testing using the Benjamini-Hochberg method for correcting the false discovery rate [36]. NC denotes *P*-value >0.05

## Discussion

The emergence of a large number of bioinformatics tools over the past decade have made it easier for researchers to interpret the vast amount of transcriptomic data generated from DNA microarrays, RNA sequencing and other gene expression methods. However, the diverse range of tools have also created a number of challenges. First, differences in assumptions underlying the methods used have made it more difficult to compare studies that have used genome-wide expression profiling to study similar pathophysiological phenomena, such as sepsis [5]. Secondly, depending on the methods used, the same gene expression dataset may result in different interpretations, thereby requiring close attention to the underlying statistical assumptions that have affected data interpretation.

The **first aim** of this study was to compare the biological interpretation resulting from two different bioinformatics tools – GSEA and IPA. Specifically, we investigated the pro-inflammatory response induced in two different model systems of meningococcal activation of human monocytes. Overall, both GSEA and IPA associated the differentially regulated genes to similar biological processes and functional groups, identifying that *N. meningitidis* and meningococcal sepsis plasma depleted for IL-10, respectively, induced genes associated with pro-inflammatory innate immune activation, including TNF-signaling, Toll-like receptor signaling, JAK-STAT-signaling, type I and type II interferon, as well as apoptosis and mitochondrial dysfunction. Some enriched gene sets in GSEA, such as KRAS signaling, allograft rejection, epithelial mesenchymal transition, and estrogen late response did not immediately appear relevant to the experimental condition and disease under study, and were, except for estrogen late response, not detected by IPA. In addition, when we focused specifically on genes differentially induced by the presence of IL-10, GSEA and IPA differed in the enrichment of biological processes and signaling pathways.

Overall, the rapid induction of pro-inflammatory genes by sepsis plasma is consistent with comparable previous studies of sepsis-induced gene expression at an early stage of the sepsis syndrome [36–40]. It should be noted that all these studies investigated gene expression in whole-blood or leukocyte isolates collected within 24 h of admission, which differs from our dataset which is generated by inducing gene expression in human monocytes elutriated from heparinized whole blood from healthy blood donors combined with sepsis plasma samples [26]. These studies have therefore the advantage of reporting data generated from leukocytes isolated directly from patients during the clinical course of sepsis. An advantage of our model is that the onset of pathogenic activation of the monocytes as well as the bacterial load is known. In most clinical sepsis studies, the exact onset of the disease is unknown. Furthermore, the initial blood samples are drawn from various time-points during the first 24 h of admission, and the bacterial load in plasma or the microbial molecules driving the innate immune reaction often remain unknown [36–40].

A few differences exist between GSEA and IPA. The first and foremost is that GSEA does not implement a filtering criteria prior to analysis, and therefore consider the expression of all the genes in the dataset. The main strength of GSEA is to identify the extent to which an a priori defined list of genes, which previous experiments have associated with biological processes or signaling pathways, is coordinately expressed in the dataset. This differs from the conventional approach to gene expression analysis in IPA, which normally implies setting a pre-defined cut-off based on a corrected or uncorrected *P-*value, and FC values, such as 1.5 and 2.0. A risk with this approach is that the investigator may unintentionally exclude relevant genes, and risks the loss of biologically relevant information which the bioinformatics tools are unable to detect [41]. In comparison, the underlying statistics of GSEA enables the detection of biological functions associated with coordinated, but subtle changes in gene expression levels.

In our case, the previous interpretation of gene expression profiling of human monocyte response to meningococcal infection, generated both in a *model system* [25] and in *a patient plasma system* [26], overlooked the large number of significantly regulated genes associated with mitochondrial dysfunction and oxidative phosphorylation. This was primarily due to the implemented filtering criteria consisting of a cut-off for statistical significance (*P* < 0.01) and FC (1.5). However, in the present study, using a filter of FDR 5% only, the IPA analysis suggested that *N. meningitidis* induces a strong down-regulation of a large number of genes associated with oxidative phosphorylation, consistent with what was identified by GSEA.

Another challenge to directly comparing GSEA with IPA is that the gene sets in GSEA and canonical pathways in IPA do not directly correspond to each other. The gene sets in GSEA are curated based on gene expression patterns identified in previously conducted experimental conditions. In many cases, the gene sets correspond to a broadly defined biological process (such as “inflammatory response”, “complement” or “coagulation”), or are named after the response to a particular stimulus (such as interferon-gamma or TNF). In comparison, the canonical pathways in IPA delineate more detailed biochemical steps known to result from specific exposures or receptor-binding, or describe mechanistic interactions occurring in specific diseases (refer to Panel 1). Furthermore, IPA does not limit itself to interactions observed in gene expression studies, which is the case for the hallmark gene sets from the Molecular Signatures Database [34] used for the present study. Another advantage is that IPA is also more frequently updated.

We used these two methods in a complementary manner. The Gene Set Enrichment Analysis, using the hallmark gene sets or other gene sets from the Molecular Signatures Database [34] when relevant, produced an unbiased, initial analysis of the extent to which there was coordinated expression of genes related to defined biological functions. Verification of these results was conducted in IPA, which enabled a more detailed examination of whether the differential regulation of genes was associated with mechanistic pathways, as well as locating the position and direction of change of specific genes in pathways.

The **second aim** was to investigate whether the biological processes associated with the gene expression changes in our *model system* of meningococcal sepsis was comparable to the results found in our *patient plasma system*. GSEA suggests that the simple *model system*, consisting of human monocytes stimulated in a normal pooled plasma with wild-type *N. meningitidis* and IL-10, induces transcriptomic changes associated with similar functional groups observed by the more complex plasma from patients with meningococcal sepsis. This finding has two biological interpretations. **First**, our simple human monocyte model system appears robust for the study of cellular mechanisms and biological processes associated with the host response to *N. meningitidis*. It might thus be applicable to other infectious agents. **Second**, the gene expression changes in the *model system*, where the transcriptomic response is initiated by the treatment of monocytes with *N. meningitidis* and IL-10, are reproduced in the *patient plasma system* before and after IL-10 immunodepletion. It can therefore be suggested that the dominant inducer of gene expression in meningococcal sepsis plasma is *N. meningitidis,* and that the dominant modulator of the Nm-induced gene expression pattern is IL-10.

**Finally**, this study wanted to identify whether GSEA and IPA together could generate additional insight about the human monocyte host response to meningococcal infection. The IPA analysis, supported by GSEA, suggested that *N. meningitidis* is a strong down-regulator of genes associated with oxidative phosphorylation and mitochondrial function. The reduced expression of genes associated with mitochondrial function and oxidative phosphorylation has also been identified in white blood cells isolated from sepsis patients [42, 43], and in large animal sepsis models [40]. Tang et al. have reported the inhibited expression of genes associated with mitochondrial dysfunction in a study isolating neutrophils from sepsis patients [42]. However, their study reported that gene expression was even lower in the non-septic controls. Wong et al. used IPA and identified that genes up-regulated in neutrophils from sepsis patients enriched pathways related to mitochondrial dysfunction [40]. Two more recent studies have more specifically studied the differential regulation of genes associated with oxidative phosphorylation in sepsis patients [44, 45]. One study by Weiss et al. [45] determined the differential expression of 296 nuclear-encoded mitochondrial genes in whole blood collected within 24 h of admission from pediatric patients with septic shock compared with non-septic controls. They identified that 118 of 296 genes were differentially regulated (48 up-regulated and 70 down-regulated) in septic shock patients. Between survivors and non-survivors of septic shock, the genes cytochrome C oxidase subunit VIIb and NADH dehydrogenase flavoprotein were differentially regulated. They also identified that a sub-group of patients with higher rate of multiple-organ failure and higher mortality rate had a greater repression of nuclear-encoded mitochondrial genes, compared with two other sub-groups. Another smaller study, reported in a letter to the editor by Raman et al. [44] describes gene expression in whole-blood in previously healthy children with meningococcal septicemia at 0, 4, 8, 12, 24, and 48 h from time of admission. They identified an immediate reduction of gene expression associated with oxidative phosphorylation processes, and it continued to decrease over the 48 h, leading the authors to suggest that mitochondrial dysfunction contributed to multi-organ failure. However, the letter does not report the clinical outcomes of the patients investigated. The findings from our and previous gene expression studies are consistent with a long held view that mitochondrial dysfunction and altered oxidative phosphorylation have implications in sepsis pathophysiology [46, 47]—a theme that has recently received renewed attention [44, 48, 49]. In the context of meningococcal sepsis, the intense down-regulation of genes associated with oxidative phosphorylation indicates that *N. meningitidis* as part of a pro-inflammatory response may induce dysfunction of oxidative phosphorylation and deranged energy metabolism [50, 51]—which has also been referred to as “cytopathic hypoxia” [52]—which contributes to rapidly evolving multiple organ failure. We identified furthermore that IL-10 partially reverses the down-regulation of genes associated with oxidative phosphorylation. To our knowledge, this has not been reported in previous studies of the effect of IL-10 on gene expression induced by LPS [53–56]. We have in our previous studies shown that IL-10, in response to the meningococci, both up- and down-regulates genes associated with a broad range of cellular functions [25, 26]. The increased expression of genes associated with oxidative phosphorylation induced by IL-10 indicates that activation of IL-10-signaling may improve cellular respiration, and that this may part of IL-10’s well-known attenuation of the harmful effects of meningococcal activation.

Overall, these findings should motivate functional validation by research groups specialized in the study of mitochondrial function and the oxidative phosphorylation pathway in order to generate stronger evidence about the effect of meningococci on oxidative phosphorylation in human monocytes and possibly other immune cells, and the regulatory effect of IL-10.

We propose that our study adds three additional insights to existing studies on gene expression changes related to oxidative phosphorylation in sepsis. First, we have identified that a specific pathogen – *N. meningitidis* - is able to induce down-regulation of a large number of genes associated with mitochondrial function and oxidative phosphorylation in monocytes within 3 h. Second, meningococcal sepsis plasma immunodepleted for IL-10 induces down-regulation to a similar extent. Third, the presence of IL-10 appears to partially reverse this down-regulation, adding new knowledge to the biological effects of IL-10 in meningococcal disease and possibly in other infections activating the IL-10 signaling system.

## Conclusion

We have investigated two previously generated microarray datasets of meningococcal activation of human monocytes with two different bioinformatics tools – GSEA and IPA. We have shown that relying on a single bioinformatics tool together with arbitrarily chosen filtering criteria for data analysis may result in overlooking relevant biological processes and signaling pathways associated with genes differentially expressed between compared experimental conditions. By combining GSEA with IPA, we discovered an inhibitory effect exerted by *N. meningitidis* on genes associated with mitochondrial function and oxidative phosphorylation, and that IL-10 partially reverses this strong inhibitory effect, thereby identifying, to our knowledge, yet another area where IL-10 regulates the effect of LPS.

## Additional files

Additional file 1: Canonical - Fisher p0.05 - Plasma w. IL-10 vs low LPS plasma. (TIFF 6585 kb)

Additional file 2: GSEA report - Nm + IL-10 vs Nm. (XLS 33 kb)

Additional file 3: GSEA report - Enriched in patient plasma w. IL-10 vs IL-10 immunodepleted. (XLS 31 kb)

Additional file 4: GSEA report - Nm vs Nm + IL-10.txt. (XLS 2 kb)

Additional file 5: GSEA report - Enriched in IL-10 immunodepleted vs patient plasma w. IL-10.tx. (XLS 30 kb)

Additional file 6: GSEA report - Enriched in ctr vs Nm.txt. (XLS 32 kb)

Additional file 7: GSEA report - Enriched in low LPS plasma vs IL-10 immunodepleted. (XLS 29 kb)

Additional file 8: GSEA core enrichment - Oxphos - IL-10 immunodepleted vs low LPS plasma. (XLS 61 kb)

Additional file 9: Mitochondrial dysfunction - IL-10 immunodepleted vs low LPS plasma. (XLS 16 kb)

Additional file 10: GSEA core enrichment- Oxphos - Ctr vs Nm. (XLS 62 kb)

Additional file 11: Mitochondrial dysfunction - Nm vs ctr - FDR5. (XLS 46 kb)

## Abbreviations

GSEA: Gene set enrichment analysis
IL-10: Interleukin-10
IPA: Ingenuity Pathway Analysis
Nm: *N. meningitidis*
TNF: Tumor Necrosis Factor
