# Supplementary figures and images for: Transcriptomic data from two primary cell models stimulating human monocytes suggest inhibition of oxidative phosphorylation and mitochondrial function by N. meningitidis which is partially up-regulated by IL-10
Source: BMC Immunol. 2017 Oct 27;18:46. doi: 10.1186/s12865-017-0229-5 (PMC5659018; doi:10.1186/s12865-017-0229-5)

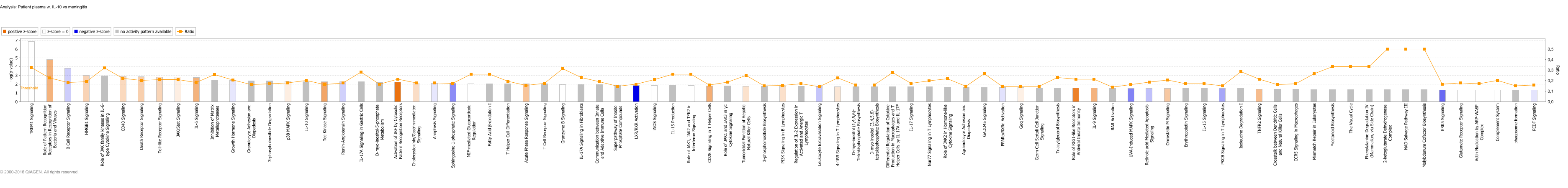

Supplement: Supplementary file 1 — Canonical - Fisher p0.05 - Plasma w. IL-10 vs low LPS plasma. (TIFF 6585 kb) [file 12865_2017_229_MOESM1_ESM.tif]
